# From the “One-Molecule, One-Target, One-Disease” Concept towards Looking for Multi-Target Therapeutics for Treating Non-Polio Enterovirus (NPEV) Infections

**DOI:** 10.3390/ph17091218

**Published:** 2024-09-16

**Authors:** Hugo Roux, Franck Touret, Pascal Rathelot, Patrice Vanelle, Manon Roche

**Affiliations:** 1 Aix-Marseille Université, CNRS, ICR UMR_7273, LPCR, Faculté de Pharmacie, 13005 Marseille, France; hugo.roux@univ-amu.fr (H.R.); pascal.rathelot@univ-amu.fr (P.R.); 2 Unité des Virus Émergents (UVE: Aix-Marseille Université, Università di Corsica, IRD 190, Inserm 1207, IRBA), 13005 Marseille, France; franck.touret@univ-amu.fr

**Keywords:** enterovirus, multi-target directed ligand, clinical trials, combination, drug design

## Abstract

Non-polio enteroviruses (NPEVs), namely coxsackieviruses (CV), echoviruses (E), enteroviruses (EV), and rhinoviruses (RV), are responsible for a wide variety of illnesses. Some infections can progress to life-threatening conditions in children or immunocompromised patients. To date, no treatments have been approved. Several molecules have been evaluated through clinical trials without success. To overcome these failures, the multi-target directed ligand (MTDL) strategy could be applied to tackle enterovirus infections. This work analyzes registered clinical trials involving antiviral drugs to highlight the best candidates and develops filters to apply to a selection for MTDL synthesis. We explicitly stated the methods used to answer the question: which solution can fight NPEVs effectively? We note the originality and relevance of this proposal in relation to the state of the art in the enterovirus-inhibitors field. Several combinations are possible to broaden the antiviral spectrum and potency. We discuss data related to the virus and data related to each LEAD compound identified so far. Overall, this study proposes a perspective on different strategies to overcome issues identified in clinical trials and evaluate the “MTDL” potential to improve the efficacy of drugs, broaden the antiviral targets, possibly reduce the adverse effects, drug design costs and limit the selection of drug-resistant virus variants.

## 1. Introduction

### 1.1. Pre-Proposal Context

Enteroviruses (EV) are a highly diverse genus of small, icosahedral viruses with single-stranded, positive-sense RNA genomes [1]. They cause a wide range of human diseases occurring most frequently in infants and young children, such as non-specific febrile illness, exanthemas, respiratory tract infections, and ocular infections. Although infections caused by non-polio enteroviruses (NPEV) are usually benign, they can be severe and have deleterious outcomes in some patients, particularly in children and immunocompromised patients.

The enterovirus genus presents seven human pathogen species (EV-A, EV-B, EV-C, EV-D and RV-A, RV-B, RV-C) divided into 11 subgroups: polioviruses (PV), coxsackieviruses groups (CVA, CVB), echoviruses (E), four enterovirus subgroups, named from A to D, and rhinovirus (RV) classified into RV-A, RV-B and RV-C subgroups. Whole enterovirus RNA strands are always composed of a coding region divided into three parts which are translated into polyproteins that are co-translationally and post-translationally cleaved by viral proteases to produce structural and non-structural proteins [2]. Viruses of the Enterovirus genus share several conserved protein motifs in the RNA-dependent RNA polymerase, capsid proteins, viral proteases, and other non-structural proteins.

The emergence of new viral types, such as enterovirus-A71 (EV-A71) and enterovirus-D68 (EV-D68), have been associated with more severe disease manifestations than previously described, including sepsis, myopericarditis, and central nervous system infections [3,4,5]. The worldwide prevalence and species distribution of enteroviruses varies among continents. For example, Enterovirus D68 is most prevalent in North America, while Enterovirus B is the predominant variant in Africa, Europe, and Southern Asia. However, there is a more variable distribution of variants among other continents, where a mixture of Enterovirus A71, Coxsackievirus A, and Enterovirus B are the most represented [6].

NPEVs can cause a wide range of health disorders with varying presentation and severity, most often in pediatric and immunocompromised patients. NPEV neonatal diseases are often related to nonspecific symptoms but can lead to severe or fatal clinical situations [7]. When considering epidemiological data, the most common pathogens with the highest incidence are due to EV-A, principally with hand, foot, and mouth disease (HFMD), whose etiologic agents are EV-A71, CVA6, CVA10, and CVA16. EV-A infections have been linked with other diseases. For example, EV-A71 or Echovirus E11 may be the causative agents of severe and life-threatening neurological symptoms—often involving long-term sequelae and cardiopulmonary complications [8]. Recently, EV-A71 has been reported to cause serious epidemics in Asia that are related to severe neurological complications like poliomyelitis [9]. Moreover, acute hemorrhagic conjunctivitis (AHC) was signaled during EV-A70 or CVA24 infections [10]. The second most represented enterovirus is EV-B, which includes Echoviruses E30, E6, E11, and Coxsackievirus B3 [6]. Severe EV-B infections have been linked mainly to acute flaccid paralysis (AFP). Meningitis or encephalitis can also be linked to EV-B infections, especially with Echovirus 30. Likewise, myocarditis is mainly induced by the CVB subgroup (from CVB1 to CVB6), and Echovirus 11 [11]. CVB1 can also cause severe infections in infants that may lead to sepsis [12]. Moreover, in 2023, at least 7 European countries experienced an outbreak of Echovirus 11, leading to serious conditions in newborns [12]. Eventually, EV-D species cause mainly pneumonia, bronchiolitis, or acute flaccid myelitis (AFM) [3]. For instance, EV-D comprises the re-emergent EV-D68, recently responsible for epidemics of respiratory diseases and polio-like paralysis AFM leading to death in some cases [13]. Interestingly, European countries have observed a re-emergence of EV-D68 post-COVID-19 lockdown [14]. Rhinoviruses have been neglected for decades because they were only related to the mild common cold with less virulence. Recently, they were recognized as critical causative agents of lower respiratory tract infections (LRTIs) and severe respiratory diseases [15,16,17].

### 1.2. The Need for Therapies

The large number of RV/EV strains and their antigenic diversity are significant obstacles in broad-spectrum vaccine development. Several clinical trials, evaluating the efficacy of small molecules, have been carried out in recent years, but there are no approved therapies targeting these viruses. Some of the reasons for these failures include viral resistance, single-targeted approaches, and lack of clinical efficacy, which pose significant obstacles in drug development [18,19]. Moreover, the importance of combination therapies involving different therapeutic mechanisms to control a disease is well-known in clinical practice. Indeed, the current treatment recommendations against hepatitis C virus (HCV) mainly involve four different combinations of Direct-Acting Antiviral Agents DAAs [20]. For instance, combinations of NS5A inhibitors and/or NS5B polymerase and/or NS3/4 polymerase inhibitors (Sofosbuvir/Velpatasvir, Glecaprevir/Pibrentasvir, Grazoprevir/Elbasvir, Sofosbuvir/Velpatasvir/Voxilaprevir) are recommended.

The treatment for HIV involves taking a combination of HIV drugs every day. Approved antiretroviral (ARV) HIV drugs are divided into eight drug classes based on how each drug interferes with the HIV life cycle. These eight classes are nucleoside reverse transcriptase inhibitors (NRTIs), non-nucleoside reverse transcriptase inhibitors (NNRTIs), protease inhibitors (PIs), fusion inhibitors, CCR5 antagonists, post-attachment inhibitors, integrase strand transfer inhibitors (INSTIs), and capsid inhibitors. Three or four classes of drugs are currently used per day according to the patient’s individual needs, the resistance profile of the virus, adverse effects, interaction with other drugs, and the previous treatment failures.

The main goal of this work is to identify promising compounds and to discuss multi-target directed ligand (MTDL) systems, from combination therapies to chimera design. The workflow is organized into two distinct parts. The first is a literature survey highlighting previous clinical trials involving NPEV inhibitors, and the second highlights selection criteria for potentially synthesized MTDL.

## 2. Results

**Scheme 1:**
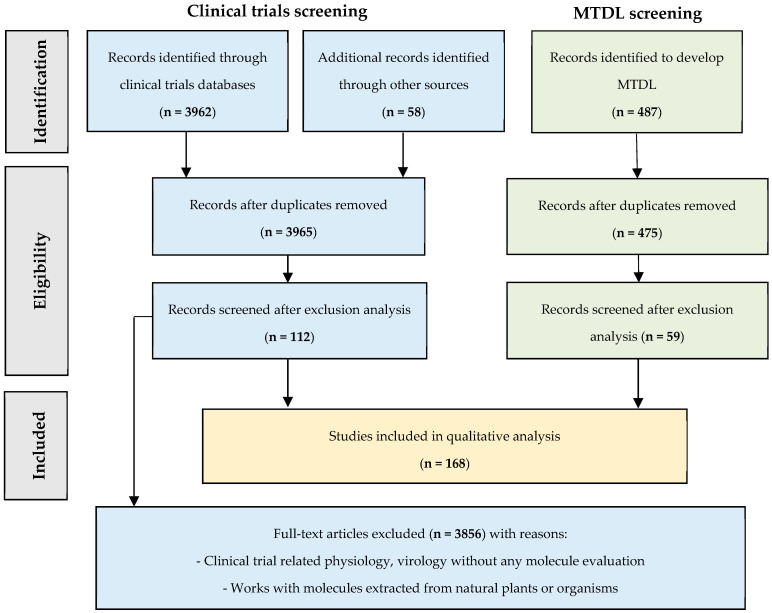
Results—Data collection [21].

A total of 3962 records were identified through the database (Clinical trials screening—3270 from CT, 25 from EudraCT, 167 from ICTRP, and 500 from PubMed). A total of 51 full clinical trials were included for analysis (55 duplicates removed and 3856 excluded). A total of 487 records were identified through PubMed (MTDL), and 59 articles were included for analysis (12 duplicates removed and 416 excluded). Fifty-eight references were added to describe the context, combinations, and biological details needed for the perspective. The results are presented in Scheme 1 [21].

Results are grouped according to antiviral drug targets in Table 1, Table 2, Table 3, Table 4, Table 5 and Table 6.

Four main types of viral targets were highlighted: structural proteins (involved in cellular entry and uncoating to release RNA), non-structural proteins (polymerase or proteases which are active during replication and translation of RNA), inhibiting viral proliferation by action on cell metabolism, and non-specified targets (unknown inhibitor mechanism of action) [22].

This section may be divided into subheadings. It should provide a concise and precise description of the experimental results, their interpretation, and the experimental conclusions that can be drawn. Two types of clinical trials are described: first, the ones where the virus was experimentally inoculated. The second type is naturally occurring cases in humans.

**Part 1: NPEV-inhibitors evaluated in clinical trials.**

### 2.1. Structural Proteins

Virus entry is the first step in the viral replication cycle and offers opportunities to develop inhibitors targeting viral structural capsid proteins [22]. Therefore, several molecules have been developed to target capsid proteins of enteroviruses.

**Figure 1:**
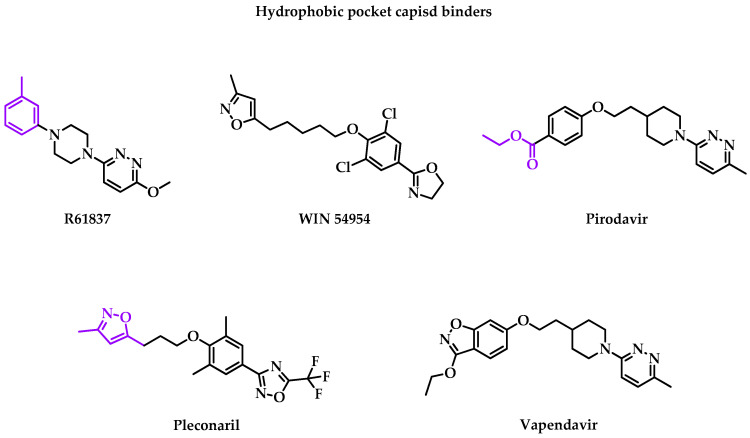
Structure of hydrophobic pocket capsid binders, which were evaluated in clinical trials. Chemical groups in purple are chemical functions that can lead to the fusion or the attachment of a linker to form an MTDL.

The most studied target is the hydrophobic pocket VP1 capsid protein constituted with two essential structures: “canyon” or “hydrophobic pocket” and “pocket factor”. Canyon is the first identified druggable surface pocket. The hydrophobic pocket is characterized by an open side, allowing accessibility for inhibitors, and a closed side, the end of the pocket. There are different binding sites in the viral capsid. When molecules bind with the capsid canyon, they stabilize the viral capsid and prevent the virus recognition by its receptors [23]. Likewise, it blocks the viral entry and thus the uncoating to release RNA and VP4 inside the cell by a higher affinity for the drug than for the receptor. The following classical hydrophobic pocket capsid binders are presented in Figure 1. Associated clinical trial parameters are presented in Table 1.

**Table 1:** Summary of clinical assays for VP1 “classical” hydrophobic pocket binders.

Publication Date	Type of Study *	Virus/Disease	Type of Infection (Time to Treatment Interval) ^†^	Drug	Pharmaceutical Form	Patient Age	Ref.
April 1989	DB PC phase II	RV-A9	Inoculation (−28 h)	R61837	Nasal spray	18–50 years old	[24]
February 1993	Rdm DB PC phase II	RV-A39	Inoculation (−42 h)	WIN 54954	Oral capsule	18–50 years old	[25]
April 1992	Rdm DB PC phase II	RV-A39	Naturally occurred (+24 h)	Pirodavir (R77,975)	Nasal spray	18–50 years old	[26]
February 1995	Rdm DB PC phase II	RV colds	Inoculation (−6/12 h)	Pirodavir (R77,975)	Nasal spray	18–65 years old	[27]
January 2000	Rdm DB PC phase II	CVA21	Inoculation (−14 h)	Pleconaril	Oral formulation	18–37 years old	[28]
15 January 2001	Phase II ^‡^	EV	Naturally occurred	Pleconaril	Suspension	2–50 years old	[29]
6 March 2002	Rdm DB PC phase II	EV Sepsis	Naturally occurred (+36 h)	Pleconaril	Oral solution/suspension	>15 days	[30]
September 2002	Clinical application	CVB	Naturally occurred (+48 h)	Pleconaril	/	<27 days	[31]
April 2003	Rdm DB PC phase II	Suspected EV meningitis	Naturally occurred (+10 days)	Pleconaril	/	<12 months	[32]
November 2005	Rdm DB PC phase III	RV common colds	Naturally occurred (+24 h)	Pleconaril	Oral tablets	>18 years old	[33]
July 2006	Rdm DB PC phase II	EV meningitis	Naturally occurred (+10 days)	Pleconaril	Oral formulation	>14 years old	[34]
31 October 2006	Rdm DB PC phase II	RV asthma & common cold	Naturally occurred	Pleconaril	Nasal spray	6–65 years old	[35]
28 May 2008	Rdm DB PC phase II	RV	Inoculation (+1/2 h)	Vapendavir	Oral capsules	NS	[36]
3 August 2010	Rdm DB PC phase II	RV	Naturally occurred	Vapendavir	/	18–70 years old	[37]
31 December 2014	Rdm DB PC phase II	RV	Naturally occurred	Vapendavir	Oral capsules	>18 years old	[38]
13 February 2015	Rdm DB PC phase II	Asthma	Naturally occurred (same day)	Vapendavir	/	18–75 years old	[39]
March 2016	Rdm DB PC phase II	EV suspected sepsis	Naturally occurred	Pleconaril	Liquid/suspension	<27 days	[40]
12 January 2017	Rdm DB PC phase II	RV Upper Respiratory	Naturally occurred (+3/5 days)	Vapendavir	Oral tablets	12–75 years old	[41]
10 November 2023	Rdm PC phase II	RV & EV COPD	Naturally occurred (+48 h)	Vapendavir	Oral tablets	40–75 years old	[33]

* Rdm = randomized; DB = double–blind; PC = placebo-controlled. ^†^ Negative delay means that the treatment began before the virus challenge or symptoms onset. The positive delay means that the treatment began after virus challenge or symptoms onset. ^‡^ Initially, the clinical trials should be placebo-controlled, but no reliable control was possible because of the rarity of EV infections. NS = not specified.

- R61837

The first clinical trial studying capsid binder as an inhibitor of enterovirus was published in 1989 for a double-blind placebo-controlled phase 2 clinical trial with an evaluation of R61837 (Janssen Pharmaceutical Ltd.) on 105 volunteers between 18 and 50 years old to treat RV-A9 infections [24]. R61837 was prepared for the nasal route, after nasal virus inoculation. The treatment began either 4 or 28 h before the virus challenge. Each patient received a 25 or 36 mg total dose of R61837 daily for 6 days. This trial concluded that R61837 doesn’t prevent RV-A9 infections but associated colds. A decrease in nasal secretion and an improvement in the clinical score were observed during the treatment but stopped when patients no longer received the drug. Patients had well-tolerated R61837. The trial conclusion was to increase the drug dose to observe higher efficacy against RV-A9.

- WIN 54954

The most famous capsid binder family is derived from drug candidates developed by Winthrop Company (WIN compounds). They possess three aromatic cores with one alkyl linker.

In 1990, a second randomized, double-blind, placebo-controlled phase 2 clinical trial was performed to evaluate the efficacy of WIN 54954 against RV-A39 infections [25]. WIN 54954 possessed an in vitro broad-spectrum activity with nanomolar potency against several RV and CV [42]. Two cohorts were recruited in Virginia and South Carolina in January and November 1990. A total of 69 volunteers between 18 and 50 years old were recruited. They were received by nasal route RV-A39. The treatment began 642 h before the virus challenge. WIN 54954 was administrated by oral capsules containing 100 or 400 mg of active ingredient for 6 days for a total dose of 1.20 or 2.40 g per day. The trials concluded that there is no significant efficacy in preventing RV-A39 colds. The main proposed limitations of this study were inadequate daily dose to have sufficient experimental power, lack of performance of measuring instruments, or development of drug resistance during the trial.

- Pirodavir

The same year, an R61837 derivative named Pirodavir (R77975) was evaluated for a randomized, double-blind, placebo-controlled phase 2 clinical trial against all RV colds [26]. Pirodavir is an antienteroviral with a nanomolar in vitro potency against a broad spectrum of rhinoviruses and several coxsackieviruses, echoviruses, and enteroviruses [43]. In the first trial, pirodavir was administrated by nasal route. Patients recruited for this study had been suffering from cold symptoms for less than 2 days, and ranged in age from 18 to 64 years. The median treatment time since the onset of illness is 24 h. Every day, patients used the nasal spray twice per nostril for 6 days. Each intranasal spray released 500 µg of drug or placebo diluted at a concentration of 5 g/L. Overall, they received 30 doses of pirodavir. The clinical trial concluded with a non-significant benefit to use the drug with efficacy and some mild adverse effects versus placebo. Indeed, there is a significantly lower frequency of virus isolation on day 3 (*p* < 0.001) and on day 5 (*p* = 0.002). However, the active cohort suffered from blood in mucus (*p* < 0.01), unpleasant taste, and nasal dryness (*p* ≤ 0.05).

A second randomized, double-blind, placebo-controlled phase 2 clinical trial was published in 1992 to evaluate the efficacy of a nasal spray containing pirodavir against inoculated RV-A39 infections [27]. The treatment began 6 or 12 h before the virus challenge. Patients between 18 and 64 years old received two sprays for each nostril per day. They received a total of 2 mg of pirodavir per day. The trial concluded that there is no significant difference in seroconversion rate. However, there is a lower overall number of days of virus shedding on days 2 (*p* < 0.001), on day 3 (*p* = 0.005) and day 4 (*p* = 0.04) for the first study. During the second and third studies, there was no significant difference between the placebo and virus-shedding cohorts. Eventually, even if the drug seems to irritate the respiratory mucosa and cause nasal dryness, there is no serious adverse effect, as blood in the mucus case reported on the active cohort.

- Pleconaril

In 1996, two phase 2 clinical trials for the compound WIN 63843 (ViroPharma, Inc., Exton, PA, USA), also named pleconaril or VP 63843, were performed to evaluate its efficacy against CVA21 infections and EV infections, respectively [28,29]. Pleconaril is a broad-spectrum inhibitor with micro or nanomolar in vitro potency against coxsackieviruses and enteroviruses, except for CVB3 [44]. It was also used to cure echovirus-infected neonates [45]. Respectively, 33 and 38 adults were enrolled to receive a 400 mg total daily dose for 7 days, or 200 mg or 400 mg three times, by the oral route. The first study concluded that pleconaril reduces viral shedding in nasal secretion (*p* < 0.001), nasal mucus production (*p* = 0.004), and total respiratory illness symptom scores (*p* = 0.013) compared with placebo after a virus inoculation. The treatment began 14 h before the virus challenge. However, it also causes some adverse effects, like nausea, abdominal pain, dysmenorrhea, and an increase in the frequency of urine. For the second trial, suspected infected children in the active cohort received three doses of 5.0 mg/kg. No delay between inclusion and treatment is reported. Pleconaril was used as compassionate/last-line treatment. Because of the rarity of EV infections, it was challenging to design a placebo-controlled trial.

In 1999, three randomized, double-blind, placebo-controlled studies were performed to evaluate pleconaril efficacy against suspected EV meningitis or sepsis [32,34,40]. These studies respectively recruited 20 sepsis suspected patients under 12 months, 250 patients over 14 years old with a white blood cell number concentration superior to 10 mm^−3^, and 61 sepsis suspected neonates (with 43 confirmed EV sepsis) patients. Pleconaril was administrated by liquid or suspended oral formulation with respective concentrations of 5 mg/kg/dose (three times a day), 200 or 400 mg (three times a day), and 8.5 mg/kg per day for 7 days. The onset of symptoms had to be, respectively, 36 h, 48 h and 10 days before administration of the first dose of study medication. Conclusions of studies on infants were encouraging with a virus culture which turned to negative quicker for the active cohort (*p* = 0.08) and a higher survival probability after the treatment (*p* = 0.26) and over 18 months (*p* = 0.07, *p* = 0.23 for validated enteroviruses infections neonates) even if the duration of hospitalization was similar (*p* = 0.18). No adverse effects were attributed to the treatment despite the drug’s tendency to accumulate. The trial on neonates finished during the study due to the expiration of the study drug. The last study performed on children and adults is more mitigated about its conclusion. Indeed, there are some limitations, such as the low number of patients with validated EV meningitis and the difficulty of oral admission for patients with nausea. The headache resolution is quicker for the active cohort with moderate or severe nausea (*p* = 0.009) but not for very severe nausea (*p* = 0.05) or without nausea (*p* = 0.15). However, there is a light low treatment-related adverse effect (*p* = 0.14) or treatment-emergent adverse effect (*p* = 0.71). The analysis is exploratory, not for licensure, due to a failure to define the clinical benefit of the Food Drug Administration (FDA).

In 2000, a randomized, double-blind, placebo-controlled phase 3 of clinical trials was performed on 2096 patients to evaluate pleconaril efficacy against RT-PCR-detected RV common colds [33]. To be included in the study, cold symptoms had to have appeared within 24 h. Patients received 200 mg of oral tablets thrice daily for 5 days. By day 3, there is no culturable virus for pleconaril-treated patients (*p* < 0.0001). By day 6, the number of samples is significantly lower for the active cohort (*p* = 0.07). The study used wild-type viruses and resistant-induced viruses. Symptom resolution of the pleconaril cohort was quicker than the placebo one if they were exposed to the wild type (*p* < 0.0001) but not favorable with an exposition to the resistance strain. Some resistance issues can explain this result.

In the early 2000s, three studies were conducted to evaluate pleconaril efficacy against enteroviral sepsis, CVB infections, both in newborns, and the common cold due to rhinovirus infections in children and adults [30,31,35]. They are either randomized, double-blind, placebo-controlled phase 2 of clinical trials or clinical application studies. Respectively, 61 were suspected of being infected, sepsis detected in four, and 311 patients receiving viral inoculations were recruited to receive the drug (respectively 5 mg/kg three times per day, 5 mg/kg per day, and 12 mg/dose twice daily) or placebo by the oral route for newborns and nasal route for children and adults. For these studies, few significant results were identified. Any result was associated or published after the end of the first trial. The onset of disease symptoms was ≤10 days prior to administration of the first medication dose. Even if it suggests a beneficial effect in combination with earlier treatment during the clinical application, no clear proof was identified that only pleconaril, as compassionate or last-line treatment, without reported delay between inclusion and treatment, decreases CVB symptoms. Finally, both drug and placebo cohorts had the same levels of RV PCR-positive colds (*p* = 0.973) in the third study, with a prophylactic use of pleconaril. As a result, no positive effect was identified in this case.

- Vapendavir

The last promising capsid binder, evaluated against enteroviruses, particularly rhinoviruses, was the compound BTA798 (Altesa Biosciences, Inc. & Biota Pharmaceuticals, Inc. & Vaxart), also named vapendavir [46]. In 2008, a randomized, double-blind, placebo-controlled phase 2 clinical trial on 240 inoculated male patients to evaluate its efficacy against the common cold [36]. The prophylactic treatment was given to subjects on day 0, 1 or 2 h after the virus challenge. Vapendavir was only once administrated by the oral route with 25, 100, or 200 mg of the drug. The result of this study identified a dose-dependent decrease in the incidence of RV-A39 infections. However, most of these infections were asymptomatic, which does not enable an evaluation of the protection against upper respiratory tract illness. The drug was well-tolerated even if one patient was excluded from the study due to neutropenic sepsis, which can be caused by the drug. This study was prematurely ended.

In 2010, a second randomized, double-blind, placebo-controlled phase 2 clinical trial was conducted to evaluate the efficacy of vapendavir against rhinoviral infections [37]. Suspected infected patients were recruited among adults, ranging from 18 to 70 years. No delay between the patient’s inclusion and treatment is reported in the accessible data. They receive twice daily a non-specified dose. The drug-treated cohort had lower cold symptoms and impact scores than the placebo cohort (*p* = 0.020).

In 2015, vapendavir was evaluated in two randomized, double-blind, placebo-controlled phase 2 clinical trials against asthma due to suspected RV infections [38,39]. For the first study, it was administered as oral capsules to 480 patients, including 24 patients over 65 years old. Each capsule contains 132 mg of the drug. Two cohorts were formed, receiving only once 264 (2 doses) or 528 mg (4 doses). These two cohorts had better clinical scores than placebo one (*p* = 0.319), individually and combined. Likewise, the 268 mg cohort had less moderate or severe asthma exacerbation (*p* = 0.563), as well as the 528 mg cohort (*p* = 0.632), or both combined (*p* = 0.942). For the second study, 455 patients aged 18 to 75 received 264 or 528 mg of drugs. The treatment began the same day as randomization. No results were associated or published after the end of the trials.

In 2017, a randomized, double-blind, placebo-controlled phase 2 clinical trial evaluated the efficacy of vapendavir against detected upper respiratory tract RV infection [41]. 65 patients, ranging from 12 to 75 years, should be recruited. The interval from symptom onset to study treatment initiation should be preferably within 72 h but up to a 5-day window is possible. Vapendavir should be administered once as tablets containing 264 mg of drugs. Unfortunately, the trial was withdrawn due to a company decision.

Eventually, a randomized placebo-controlled phase 2 clinical trial was conducted to evaluate the effect of vapendavir against COPD associated with enterovirus and rhinovirus infections [47]. Vapendavir will be administered, as soon as possible after the onset of symptoms and documentation of RV infection, but no greater than 48 h after symptom onset, twice daily as tablets containing 1000 mg (four then two daily tablets to inoculated volunteers from 40 to 75 years, for 7 days).

### 2.2. Viral Non-Structural Protein

**Figure 2:**
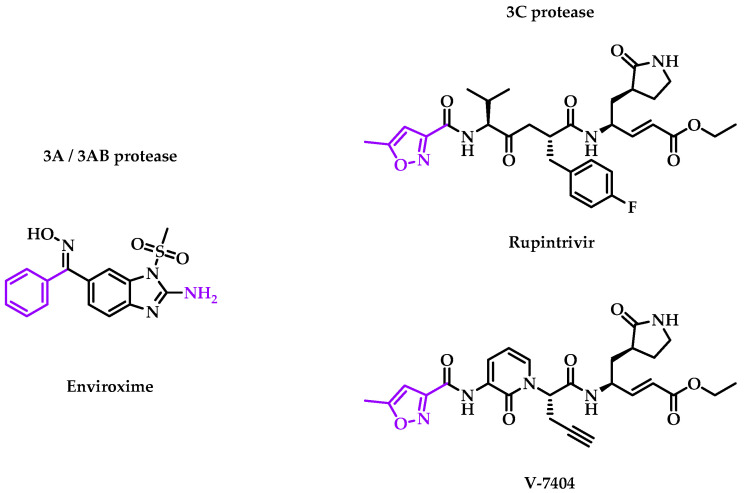
Structure of 3A/3AB and 3C inhibitors, which were evaluated in clinical trials. Chemical groups and atoms in purple are chemical functions that can lead to the fusion or the attachment of a linker to form an MTDL.

The viral capsid is the first accessible target from the viral replication cycle. However, other targets were identified during viral replication or translation. In addition to structural proteins, enteroviruses possess non-structural proteins critical to the replication cycle. Several inhibitors were evaluated in clinical trials, and their chemical structures are represented in Figure 2.

**Table 2:** Summary of clinical assays of non-structural protein inhibitors.

Publication Date	Type of Study *	Virus/Disease	Type of Infection (Time to Treatment Interval) ^†^	Drug	Target	Pharmaceutical Form	Patient Age	Ref.
20 July 1981	PC phase II	RV-A9	Inoculation (−24 h)	Enviroxime	3A/3AB protein	Nasal spray	18–50 years old	[48]
July 1982	Rdm DB PC phase II	RV-A39	Inoculation (−24 h)	Enviroxime	3A/3AB protein	Nasal spray/oral	18–36 years old	[49]
December 1982	Rdm DB PC phase II	RV	Inoculation (−24 h)	Enviroxime	3A/3AB protein	Nasal spray	NS	[50]
July 1983	DB PC phase II	RV-A9	Inoculation (−24 h)	Enviroxime	3A/3AB protein	Nasal spray	18–50 years old	[51]
January 1985	Rdm DB PC phase III	RV common cold	Inoculation (−44 h)	Enviroxime	3A/3AB protein	Nasal suspension	8–65 years old	[52]
February 2002	Rdm DB PC phase I	RV	/	Rupintrivir	3C protein	Nasal spray	18–50 years old	[53]
December 2003	Rdm DB PC phase II	RV-A39, RV-A21	Inoculation (−6/+24 h)	Rupintrivir	3C protein	Nasal spray	18–60 years old	[54]
July 2005	Phase I	RV	/	V-7404	3C protein	Oral formulation	18–55 years old	[55]
17 September 2021	Rdm DB PC phase I	EV	/	V-7404	3C protein	Oral solution	18–45 years old	[56]

* Rdm = randomized; DB = double–blind; PC = placebo-controlled. ^†^ Negative delay means that the treatment began before the virus challenge or symptoms onset. The positive delay means that the treatment began after the virus challenge or symptoms onset. NS = not specified.

#### 2.2.1. 3A/3AB Proteases

Enteroviral 3A protease was described as a promotor of binding between EV and the host cell phosphatidylinositol 4-kinase III β (PI4KIII β) by its interacting with host factor ACBD3 [57]. Indeed, the replication of EV-A71 may depend on PI4KIII β even if the mechanism is not defined to date [58,59,60]. Thus, 3A protease seems to play a key role in replicating EV-A71 and EV-D68, but not for RV-A16. Enteroviral 3A protease comes from 3AB protease, which is cleaved by 3C protease [61]. The role of 3AB protease in EV replication is not well known. However, some RNA chaperone activity was identified for EV-A71 [62]. The protein recruits viral and host factors to form a replication complex important for viral replication. Both 3A protease and its precursor, 3AB protease, have membrane-binding properties.

- Enviroxime

Enviroxime (Lilly Research Laboratories, Indianapolis, IA, USA) is an inhibitor of EV and RV 3A /3AB protease [63]. It was one of the first anti-enteroviral compounds with a determined mechanism, which was studied in phase 2 clinical trials. In 1981, a placebo-controlled and a randomized, double-blind placebo-controlled study was published evaluating the efficacy against RV-A9 infections and RV-A39 infections, respectively [48,49]. Enviroxime was administrated by nasal route, as well as the inoculated virus. The treatment began 1 day before the virus challenge. 48 and 40 adults received 102 mg, four times daily for 6 days and one or three times daily for 8 days, respectively. The first study concluded that a decrease in mean daily nasal secretion weights was observed in the active cohort (*p* = 0.02). However, there is no significant difference in clinical score between cohorts (*p* = 0.28) or viral quantity in nasal washings (*p* = 0.16). A slightly substantial difference from the third day for rhinorrhea was observed. There is a negative correlation between quantitative indices of RV infections and drug quantity. Enviroxime was well tolerated except for one patient who presented nausea due to the active ingredient. The second study measured no significant protection against RV-A39 infections versus placebo.

In 1982, two randomized, double-blind, placebo-controlled, phase 3 and phase 2 clinical trials were performed against natural RV common cold and general rhinovirus infection, respectively [50,51]. The two studies gathered 1020 patients with signs of infection and 99 inoculated patients to receive 42 mg (four then two doses per day) and 16 mg (a daily dose) weekly of enviroxime by nasal route. The treatment began 1 day before the virus challenge. The first study concludes that there is limited benefit due to the need for clear reproducibility of the administration or the little practical importance of delivering media. Unlike these first conclusions, most of the results of the second study were identified as negative for the drug administration.

Finally, a last double-blind placebo-controlled phase 2 clinical study evaluating enviroxime against inoculated RV-A9 infection was published in 1983 [52]. The treatment began 44 h before virus challenge. A total of 41 patients were recruited, ranging from 18 to 64 years, to receive 284 µg of drug per nostril, six times a day, for 5 days, as a nasal spray solution. The mean clinical score of enviroxime-treated patients was significantly lower than the placebo cohort on day 5 (*p* = 0.04). However, no differences were identified in nasal secretions in both cohorts.

#### 2.2.2. 3C Protease

As described previously, 3C protease cleaves the viral polyprotein at eight sites, including those to release 3AB and 3A proteases [61]. Due to the role 3C protease plays in EV processing, it makes the protease an essential target against EV. Indeed, 3C-protease inhibitors tend to be more effective than enviroxime and pleconaril, which were more effective than vapendavir and pirodavir [64]. The 3C protease is a protease found in picornaviruses and performs several functions. In coxsackieviruses, 3C protease activates apoptosis via several caspases (caspase-3, caspase-8, and caspase-9) [65]. Moreover, in rhinoviruses, 3C protease targets OCT-1 transcription factor for proteolytic cleavage [65,66]. These aspects make this a target to be favored. Promising molecules have been evaluated in clinical trials since discovering the role of 3C protease inhibitors.

- Rupintrivir

The historic 3C inhibitor of enterovirus is rupintrivir (Agouron Pharmaceuticals, Inc., San Diego, CA, USA), first synthesized in 1999 [67]. Three years later, a randomized, double-blind, placebo-controlled phase 1 clinical trial was published to evaluate the pharmacokinetic parameter of the RV inhibitor [53]. The drug was delivered to 36 male patients, ranging from 18 to 50 years, as a nasal spray solution containing 4 or 8 mg, one or six times per day, for 7 days. Rupintrivir was measured with low concentration (<0.52 ng/mL in plasma and <2% in nasal wash) at the end of treatment. Few metabolite concentrations were also observed. However, rupintrivir was identified as safe and well tolerated over time, with substantial drug detection for 9 h after administration.

In 2003, a randomized, double-blind, placebo-controlled phase 2 clinical trial was published to evaluate the efficacy of rupintrivir against RV-A39 and RV-A21 infections [54]. The virus was administered to patients as nasal drops. A dose was given 6 h prior to the viral challenge for the prophylaxis study and 24 h after for the treatment. A total of 8 mg of the drug was delivered two or five times a day for 7 days to 202 patients (83 males and 119 females), ranging in age from 18 to 60 years, as a nasal spray solution. The active cohort had lower mean total daily symptoms than the placebo cohort (*p* = 0.014) and a higher incidence reduction on viral culture (*p* = 0.04–2 doses per day group/*p* = 0.03–5 doses per day group). It also has a decrease in viral titers (*p* < 0.05) but no significant difference in cold cases. The mean cumulative nasal discharge was lower for drug-treated patients (*p* = 0.05–2 doses per day group/*p* = 0.12–5 doses per day group). Finally, no significant local nasal adverse effects were declared.

- V-7404

In 2005, a phase 1 clinical trial was published to evaluate the pharmacokinetic parameters of anti-rhinoviral V-7404 (Pfizer Global Research and Development, San Diego, CA, USA), a rupintrivir derivative, administered in one single dose orally for fasted or fed patients [55,68]. The oral route has more risk of adverse effects than the nasal route but can access other RV replication sites for diseases localized elsewhere than in the respiratory tract. A total of 14 male patients, ranging from 18 to 50 years, received 200, 500 (fasted or fed), 1000, or 2000 mg of drug. The study concluded that for 500 mg doses, the free maximum serum concentration (C_max_) was superior to the compound’s half maximal effective concentration (EC50), inhibiting 80% of RV serotypes.

In 2018, a randomized, double-blind, placebo-controlled phase 1 clinical trial was conducted to evaluate the V-7404 safety and pharmacokinetic parameters against EV infections [56]. For this study, 77 patients (33 males and 44 females) aged 18 to 45 years were recruited to receive an oral solution. Different weights (200, 500, 1000, and 2000 mg) were tested in one single dose or one or two doses per day for 2 weeks. V-7404 was well tolerated, even with multiple doses, and with acceptable safety. Only mild or moderate treatment-emerged adverse effects were observed. The drug was adsorbed in less than an hour, and its half-life took between 0.3 and 2.3 h. The plasma concentration of the drug was quickly under detection, which was the limit of measurement tools. This is why an exponential decrease in its plasma concentration was observed. Pharmacokinetic parameter quality increases with a dose-proportional factor. At last, fed patients have a longer time to reach maximum serum concentrations than fasted ones.

### 2.3. Targeting Human-Coded Protein Promoting Enterovirus Replication

**Figure 3:**
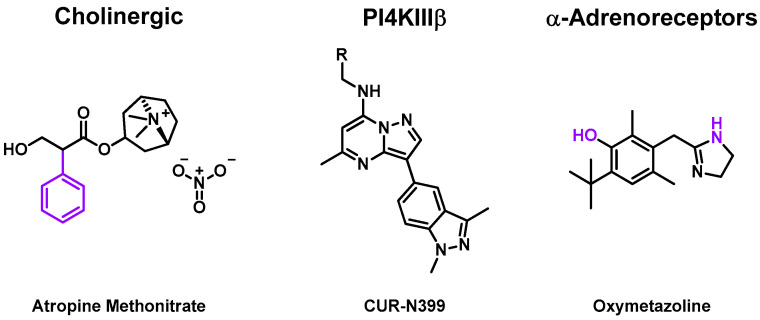
Structure of cholinergic, phosphatidylinositol 4-kinase beta (PI4KIII β), α-adrenoreceptor inhibitors, which were evaluated in clinical trials. Chemical groups and atoms in purple are chemical functions that can lead to the fusion or the attachment of a linker to form an MTDL.

Some molecules also target human enzymes, and human-coded proteins, which imply a reduction of viral charge and try to avoid any major side effects. They target cell metabolism functions that enteroviruses use to replicate. Inhibiting these steps needs to balance the viral activity reduction and the cell’s vital task. Some compounds, represented in Figure 3, were evaluated in several clinical trials (Table 3).

**Table 3:** Summary of clinical assays of drugs targeting cell metabolism.

Publication Date	Type of Study *	Virus/Disease	Type of Infection (Time to Treatment Interval) ^†^	Drug	Target	Pharmaceutical Form	Patient Age	Ref.
January 1987	Rdm DB PC phase II	RV-A39	Inoculation (−1 day)	Atropine Methonitrate	Anticholinergic	Nasal spray	>18 years old	[69]
23 July 2020	Rdm DB PC phase III	EV, RV	Inoculation	CUR-N399	PI4KIII β	Oral capsule	>18 years old	[70]
September 2010	Rdm DB PC phase II	RV-A39	Inoculation (−3 h)	Oxymetazoline	α-Adrenoreceptor	Intranasal liquid	18–65 years old	[71]

* Rdm = randomized; DB = double–blind; PC = placebo-controlled. ^†^ Negative delay means that the treatment began before the virus challenge or symptoms onset.

#### 2.3.1. Cholinergic Receptors

Cholinergic receptors play a significant role in signal transduction in the nervous system [72]. In 1987, randomized, double-blind, placebo-controlled phase 2 clinical trials were published to evaluate the efficacy of atropine methonitrate (Vick’s Research Center, Shelton, CT, USA), a known cholinergic antagonist, against RV-A39 infections in inoculated patients [69,73]. The treatment began 1 day before virus challenge. Even if the cholinergic mechanisms in common colds were not clearly defined, the study recruited 30 adults to receive in one time two injections of nasal spray solution per nostril containing 250 or 500 µg of the drug. Patients had to repeat the treatment four times per day for 5 days. No significant effects on nasal symptoms in the active cohort were observed, but nasal mucus production was reduced for 250 µg-treated patients with a four-daily prescription. However, for this concentration assay, the placebo weight of nasal mucus in the placebo cohort was higher than for other concentrations. At last, for high doses of drugs, some nasal adverse effects were detected.

#### 2.3.2. Phosphatidylinositol 4-Kinase Beta (PI4KIII β)

As previously described, PI4KIII β plays a critical role in EV replication, including EV-A, EV-D and CVB [74]. EV binding with PI4KIII β leads to a change of a cellular in endomembranes to build replication organelles, which protect EV RNA during the genome replication. In 2021, a randomized, double-blind, placebo-controlled phase 1 clinical trial was begun to assess the efficacy of CUR-N399 (Curovir AB) against chronic obstructive pulmonary disease (COPD) due to inoculated enterovirus or rhinovirus infections [70]. The delay between treatment initiation and virus challenge was not reported. CUR-N399 is a PI4KIII β inhibitor, especially against EV-A71 infections [75]. Oral capsules were distributed to 74 adults having either a drug or placebo inside. Two types of study were decided. The first delivered one dose to patients containing 2.5, 7.5, 17.5, 35 or 50 mg. The other was a multiple-dose study. For 7 days, patients received 10, 25, or 50 mg per day. Currently, the results have not been published.

#### 2.3.3. α-Adrenoceptor

α-Adrenoceptors are divided into two subtypes (α1-adrenoceptors and α2-adrenoceptors) with a large panel of effects on human cell behavior. Contrary to β-adrenoceptor, they interact globally for smooth muscle contraction, glycogenosis, adenylyl cyclase inhibition, intracellular cAMP reduction, neurotransmitter release, or central vasodilation reduction [76,77]. It was shown that oxymetazoline reduces in vitro expression of the intracellular adhesion molecule (ICAM) 1 receptor, which is the major receptor used by RV to enter human cells. In 2007, oxymetazoline, an α-adrenoceptor agonist, was used in a randomized, double-blind, placebo-controlled phase 2 clinical trial against inoculated RV-A39 infections [71,78]. The treatment began 3 h after the virus challenge. It was used for many years to relieve nasal congestion and obstruction during common colds. It was shown that oxymetazoline reduces in vitro expression of the intracellular adhesion molecule (ICAM) 1 receptor, which is the major receptor used by rhinoviruses to enter human cells [83]. The drug is administrated as an intranasal liquid solution containing 22.5 µg of drug diluted in 45 µL of citrate buffer three times a day for 5 days. A total of 94 patients (45 males and 49 females), ranging from 18 to 64 years, were recruited to assess the study. On the second day of treatment, a lower virus titer was observed in the active cohort (*p* = 0.04). No medication-caused severe adverse effects were identified except 2, one for the active cohort and one for the placebo.

### 2.4. Unknown or Other Mechanisms of Action

In addition, some inhibitors of enterovirus were developed without identification of the intended target or because they were like other pathogens (viruses, bacteria, etc.) and reduced symptoms from close diseases.

#### 2.4.1. Anti-Rhinovirus

**Figure 4:**
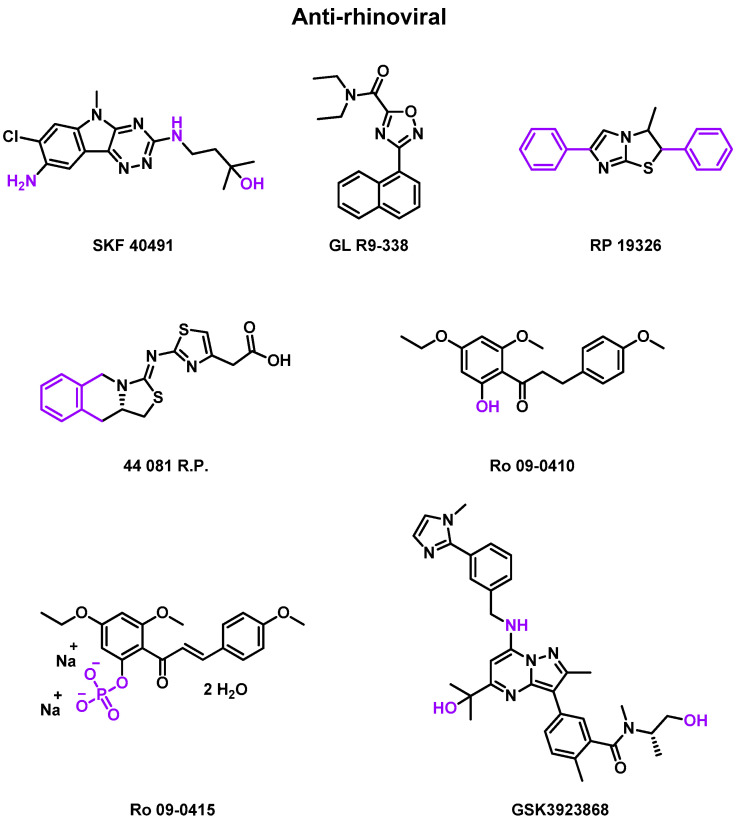
Structure of anti-rhinoviral compounds, which were evaluated in clinical trials. Chemical groups and atoms in purple are chemical functions that can lead to the fusion or the attachment of a linker to form an MTDL.

In the 1950s, some anti-rhinovirals were developed without identifying the viral part targeted and evaluated in clinical trials (Table 4) to assess their efficacy, as represented in Figure 4. Clinical trials were performed using nasally administered rhinoviruses.

**Table 4:** Summary of clinical assays of drugs with unknown mechanism of action against Rhinoviruses.

Publication Date	Type of Study *	Virus/Disease	Type of Infection (Time to Treatment Interval) ^†^	Drug	Pharmaceutical Form	Patient Age	Ref.
July 1976	Rdm DB PC phase II	RV-B3	Inoculation (−1 day)	SKF 40491, GL R9-338, RP 19326	Solution spray/suspension drop	18–50 years old	[79]
October 1984	Rdm DB PC phase II	RV-A9	Inoculation (−1 day)	Ro 09-0415	Oral capsule	18–50 years old	[80]
May 1985	Rdm PC phase II	RV-EL, RV-1B	Inoculation (−1 day)	44 081 R.P.	Nasal spray	18–50 years old	[81]
December 1987	DB PC phase II	RV-A2	Inoculation	Ro 09-0410	Nasal spray	>18 years old	[82]
July 1990	Phase II	RV-A2	Inoculation	Ro 09-0410	DMSO solution	18–50 years old	[83]
13 May 2022	Rdm DB PC phase I	Asthma with RV	Inoculation	GSK3923868	Inhalation powder	18–65 years old	[84]
26 May 2022	Rdm DB PC phase I	COPD with RV	Inoculation	GSK3923868	Inhalation powder	18–65 years old	[85]

* Rdm = randomized; DB = double–blind; PC = placebo-controlled. ^†^ Negative delay means that the treatment began before the virus challenge or symptoms onset.

- Rhône Poulenc (RP) studies

Rhône Poulenc was one of the first laboratories to synthesize some anti-rhinoviral candidates evaluated in clinical trials. In 1976, a randomized, double-blind, placebo-controlled phase 2 clinical trial was published to evaluate the efficacy of SKF 40491 (Smith Kline and French Laboratories, Philadelphia, PA, USA), GL R9-338 (Glaxo Laboratories, Greenford, England), and RP 19326 (Rhône Poulenc, Vitry-sur-Seine, France) against RV infections [79]. The treatment began 1 day before the virus challenge. As a solution spray or a suspension drop, these compounds were delivered to 89 patients with one dose of 1, 6.8, and 15 mg per day respectively. Unfortunately, the in vivo half-time of these drugs was too short to observe striking clinical improvement.

Rhône Poulenc conducted another randomized placebo-controlled phase 2 clinical trial in 1987 to evaluate the effect of 44 081 R.P. (Rhône Poulenc, Vitry-sur-Seine, France) against inoculated RV-EL and RV-1B infections [81]. The treatment began 1 day before the virus challenge. A total of 58 patients were recruited to receive 300 µg of the drug or placebo six times daily for 6 days. Again, no significant efficacy was observed against RV infections.

- Roche (Ro) studies

Roche also performed three phase 2 clinical trials to measure the efficacy of one different compound each time against RV infections. In 1984, the first randomized, double-blind, placebo-controlled study was published to evaluate the effect of Ro 09-0415 (Nippon Roche KK, Kamakura, Japan) against inoculated RV-A9 infections [80]. The treatment began 15 h before the virus challenge. The drug was delivered as an oral capsule, containing 100 mg of the drug, twice daily, for 6 days, to a part of 57 patients. The study concluded with no significant prevention or decrease in the severity of the infection. This can be explained by the compound’s too-low concentration reaching the nasal mucosa. Moreover, the treatment formulation seemed to be an irritant when used intranasally.

In 1987, a Ro 09-0410 (Nippon Roche KK, Kamakura, Japan & Roche Research Laboratory, WeLyn Garden City, United Kingdom) dephosphorylated Ro 09-0415 against RV-A2 infections report study was published [82]. Ro 09-0410 inhibits RV uncoating [86]. A total of 50 mg of the drug was sprayed in 0.05 mL of nasal solution, three times per nostril and three times daily, for 5 days. This double-blind placebo-controlled study recruited 50 adults. No prevention of viral infections was observed, but there was a tendency to lower the virus shedding and the antibody response and increase the nasal secretion (0.01 < *p* < 0.05).

Finally, in 1990, another phase 2 clinical trial was published, evaluating the effect of RV-A2-resistant at the same drug (Ro 09-0410) delivered this time in an oral solution [83]. 42 patients were recruited. The main goal of this study was to evaluate the effect of wild-type viruses versus resistant-induced viruses. Thus, no in vivo evaluation of the drug was performed.

- GlaxoSmithKline (GSK) studies

Finally, GlaxoSmithKline’s laboratory is conducting two randomized, double-blind, placebo-controlled phase 1 clinical trials, which began in 2022, for its compound GSK3923868 (GlaxoSmithKline Research & Development Limited, London, United Kingdom) against asthma and chronic obstructive pulmonary disease (COPD) during rhinovirus infections [84,85]. The treatment began after a viral challenge. Both studies recruited 68 patients, ranging from 18 to 64 years, divided into two cohorts, where the active cohort received the drug as an inhalation powder.

#### 2.4.2. Broad Spectrum Antiviral

**Figure 5:**
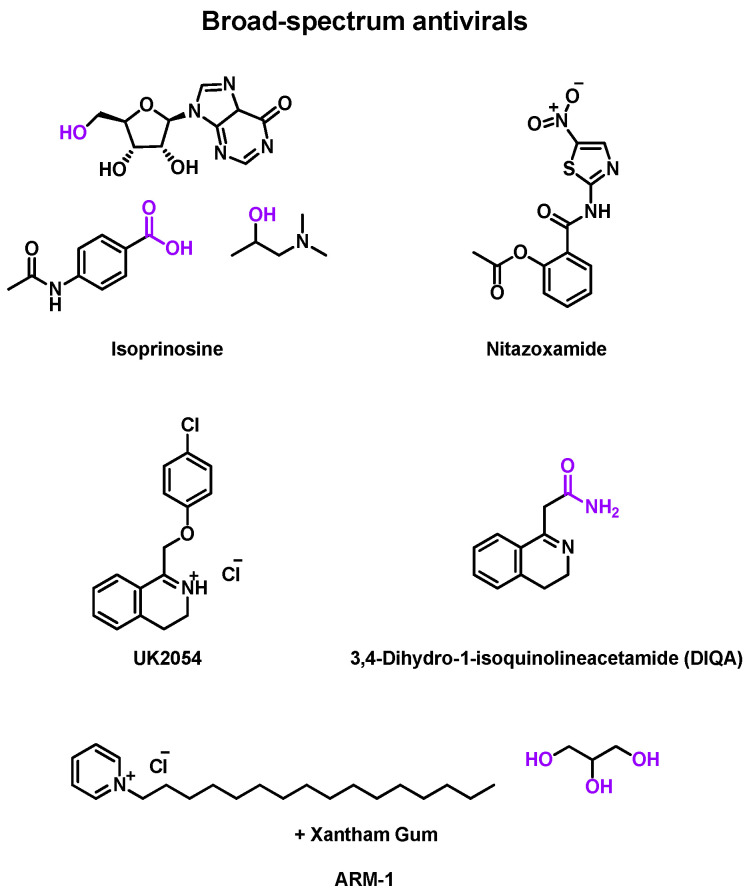
Structure of broad-spectrum antiviral compounds, which were evaluated in clinical trials. Chemical groups and atoms in purple are chemical functions that can lead to the fusion or the attachment of a linker to form an MTDL.

Numerous rhinovirus inhibitors were developed and studied in clinical trials. However, many other broad-spectrum antivirals have also been evaluated against EV infections (Table 5) and other upper respiratory tract infections caused by viruses. They are represented in Figure 5.

**Table 5:** Summary of clinical assays of broad-spectrum antiviral against Enteroviruses.

Publication Date	Type of Study *	Virus/Disease	Type of Infection (Time to Treatment Interval) ^†^	Drug	Pharmaceutical Form	Patient Age	Ref.
March 1973	DB PC phase II	RV-A9, RV-A13	Inoculation (−1 day)	Isoprinosine	Oral tablet	18–50 years old	[87]
April 1974	DB PC phase II	RV-A32, RV-B44	Inoculation	Isoprinosine	Oral tablet	25–48 years old	[88]
March 1977	Rdm DB PC phase II	RV-A21	Inoculation	Isoprinosine	Oral tablet	>18 years old	[89]
18 July 2018	Rdm DB PC phase III	EV, RV	Naturally occurred (+40 h)	Nitazoxanide	Oral tablets	>12 years old	[90]
13 May 2020	Rdm DB PC phase III	EV, RV	Naturally occurred (+72 h)	Nitazoxanide	Oral tablets	>12 years old	[91]
May 1970	Rdm DB PC phase II	RV-A9	Inoculation (−1 day)	UK2054	Oral formulation	NS	[92]
December 1973	DB PC phase II	RV-A24	Inoculation (−1 day)	DIQA	Oral capsule	21–42 years old	[93]
14 January 2017	Rdm DB PC phase II	Upper respiratory	Naturally occurred	ARM-1	Oral spray	18–43 years old	[94]

* Rdm = randomized; DB = double–blind; PC = placebo-controlled. ^†^ Negative delay means that the treatment began before the virus challenge or symptoms onset. The positive delay means that the treatment began after the virus challenge or symptoms onset. NS = not specified.

- Isoprinosine (Inosiplex^®^)

For instance, isoprinosine (Newport Pharmaceuticals, Newport Beach, CA, USA), a combination of inosine, acetamidobenzoic acid, and dimethylaminoisopropanol, has a broad spectrum against several viruses [95]. Three double-blind, placebo-controlled phase 2 clinical trials were published between 1973 and 1977 to evaluate the effect of isoprinosine delivered as an oral tablet against inoculated viruses. The treatment for all trials began 1 day before the virus challenge. The first study targeted RV-A9, and RV-A13 [87]. 55 patients were recruited. They received 6 g daily for 7 days. No significant differences were identified between placebo or drug treatment [96].

In 1974, another clinical trial focused on RV-B30 and RV-B44 infections [88]. A total of 37 males received 1.5 g of the drug or placebo four times. A slight clinical improvement was observed for drug-treated patients, but it was not significant enough to prove the drug’s efficacy on these RV serotypes [96].

Finally, a third randomized study was published to evaluate the effect against RV-A21 [89]. A total of 39 patients were recruited to receive oral tablets. In the active cohort, patients received daily 4 g of the drug for 5 or 7 days. This time, significant reductions in clinical illness and viral shedding were observed [96]. Indeed, the active cohort had a lower mean symptom score (*p* = 0.04) or cumulative symptoms score (*p* < 0.001). However, no significant difference was identified in mean symptom durations (0.05 < *p* < 0.1).

Consequently, isoprinosine is active against RV-A21 and slightly effective against RV-B30 and RV-B44 but not against RV-A9 and RV-A13. No adverse effects caused by the drug were identified. Clinical trials with more patients in cohorts could clarify the efficacy of this drug against RV infections.

- Nitazoxanide

Another actor developed a compound series of broad-spectrum antivirals. In 2018, a randomized, double-blind, placebo-controlled phase 3 clinical trial was performed to evaluate the efficacy of nitazoxanide (Romark Laboratories L.C.) against EV and RV infections [90]. A total of 1756 suspected infected adults were recruited to receive 300 mg of the drug twice daily for 5 days. The onset of illness was no more than 40 h before enrollment in the trial. Here, the onset of illness is defined as the first time at which the subject experienced rhinorrhea, cough, sore throat or nasal obstruction. The drug is administered by oral tablets. A slight improvement in infection management was observed in the active cohort. For instance, it takes a quicker time from the first dose to symptom response (*p* = 0.4009) or time to regain the ability to perform normal activities (*p* = 0.1923) than the placebo cohort. Likewise, less complications were observed for drug-treated patients (*p* = 0.2057). Eventually, they also had a lower proportion of positive for EV/RV RT-PCR for day 7 (*p* = 0.0132). In 2020, a second phase 3 clinical trial began under the same conditions as the previous study, adding a vitamin super B-complex administered to 800 suspected infected adults with either drug or placebo [91]. The onset of symptoms should be no more than 72 h before enrollment in the trial. In that case, the onset of symptoms is defined as the earlier of the first time at which the subject experienced subjective fever or any respiratory symptom (head, throat, nose, chest, or cough symptoms). Currently, this study is still recruiting patients. This clinical trial can be considered as a change of formulation in the first trial.

- Others

Some other laboratories submitted several antivirals to evaluate their efficacy against upper respiratory or RV infections. In 1963, a randomized, double-blind, placebo-controlled phase 2 clinical trial was performed to measure the effect of UK2054 (Messrs Pfizer Ltd., Shibuya City, Japan) against inoculated RV-A9 infections [92]. The treatment began 1 day before the virus challenge. 60 patients were divided into two equal cohorts. No antiviral activity was found in this trial.

In 1973, a double-blind, placebo-controlled phase 2 clinical trial was published to assess the effect of 3,4-dihydro-1-isoquinolineacetamide (DIQA) (Hoffmann-La Roche, Inc., Nutley, NJ, USA) against inoculated RV-A24 infection [93]. The treatment began 1 day before the virus challenge. Among 21 male volunteers, ranging in age from 21 to 42, 10 received 500 mg of the drug at one time in an oral capsule. No significant difference in cold reduction was observed even if the active cohort’s symptoms were milder (rhinorrhea).

Finally, in 2013, a randomized, double-blind, placebo-controlled phase 2 clinical trial was conducted to evaluate the efficacy of ARM-1 (ARMS Pharmaceutical LLC/Oasis Consumer Healthcare) in preventing upper respiratory, including rhinovirus infections [94]. ARM-1 is a formulation of cetylpyridinium chloride, mixed with glycerin and xanthan gum, already identified as an antiviral [97]. The study recruited 94 healthy patients (48 males and 46 females) aged 18 to 43 years to receive oral spray solutions. As a prophylactic treatment, no delay between symptom onset and treatment initiation was reported. This spray contained either a placebo or a drug. Patients sprayed three doses, three times daily, for 75 days. A lower detected upper respiratory infection (*p* = 0.41) and cough symptom (*p* = 0.012) were compared to the placebo cohort in the active cohort. Between days 5 and 9, drug-treated patients had a lower duration of non-fever symptoms (*p* = 0.019). The drug seemed to be safe and well-tolerated, with a reduction of symptoms and no drug-related adverse effects. However, some limitations were identified, such as the slight power of the drug in URI incidence and the lack of patients with symptoms. Future studies may be performed on multiple sites and seasons for more significant results.

#### 2.4.3. Other Drug Repurposing

**Figure 6:**
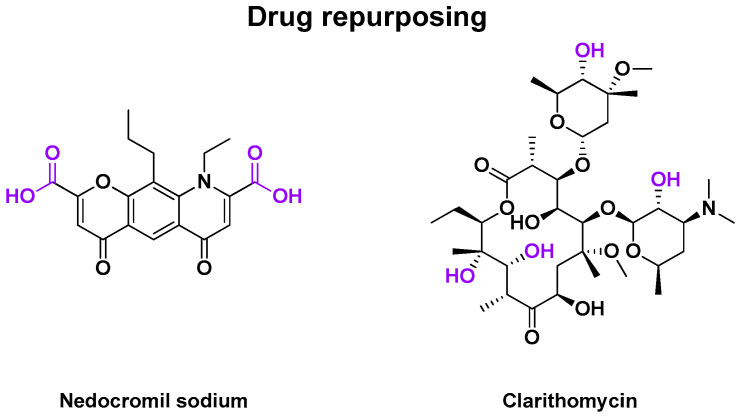
Structure of drug repurposing for antiviral compounds, which were evaluated in clinical trials. Chemical groups and atoms in purple are chemical functions that can lead to the fusion or the attachment of a linker to form an MTDL.

Eventually, some drug repurposing led to phase 2 clinical trials against RV infections. They are described in Table 6. In 1990, a randomized, double-blind, placebo-controlled study to evaluate the effect of nedocromil sodium (Fisons pic, Pharmaceutical Division), an anti-asthmatic compound represented in Figure 6, against RV-A9 and RV-B14 infections was published [98,99]. The virus was nasally administered to human volunteers. The treatment began 1 day before the virus challenge. A total of 49 patients were recruited to receive a placebo or drug as a nasal spray solution. The active cohort was exposed to 1.3 mg of the drug in 0.13 mL per nostril, four times daily, for one week. The study concluded with a lower nasal secretion for drug-treated patients (*p* < 0.05). Assays of drug tolerance identified no risk for nedocromil sodium with no significant local irritation. However, no difference was observed in the frequency of viral shedding efficacy with no activity in vitro.

In 2000, a second drug repurposing led to a randomized, double-blind, placebo-controlled study evaluating the efficacy of clarithromycin against RV-A16 infections [100]. Clarithomycin is an antibiotic for treating pulmonary diseases or skin and ORL infections [101]. Its structure is represented in Figure 6. The virus was inoculated into patients. The treatment began 1 day before the virus challenge. Treatment was delivered as two daily oral capsules, each containing 500 mg of the drug or 160/800 mg of control (trimethoprim-sulfamethoxazole), for 8 days. A total of 24 adults were recruited and divided into two cohorts. The drug-treated patients had no clinical effects compared to the control antibiotic cohort.

**Table 6:** Summary of clinical assays of other drugs against Enteroviruses.

Publication Date	Type of Study *	Virus/Disease	Type of Infection (Time to Treatment Interval) ^†^	Drug	Type of Drug	Pharmaceutical Form	Patient Age	Ref.
January 1990	Rdm DB PC phase II	RV-A9, RV-B14	Inoculation (−1 day)	Nedocromil sodium	Mast cell stabilizer	Nasal spray	18–50 years old	[98]
15 April 2000	Rdm DB PC phase II	RV-A16	Inoculation (−1 day)	Clarithromycin	Antibiotic	Oral capsule	>18 years old	[100]

* Rdm = randomized; DB = double–blind; PC = placebo-controlled. ^†^ Negative delay means that the treatment began before the virus challenge or symptoms onset.

**Part 2: Filters to obtain an effective broad-spectrum anti-enterovirus drug.**

### 2.5. Candidates’ Selection for MTDL Strategy

Several criteria must be evaluated to select candidates for MTDL design. One of the first is the number of enteroviruses inhibited by the compound. For instance, some capsid binders are not effective anymore against RV-C, due to modified conformation of the VP1 capsid protein compared to other EVs [102,103,104]. However, combining different compounds in an MTDL can be complementary to target all the enterovirus of interest. Another main criterion to consider is the inhibitor’s potency against viruses, either in vitro or in vivo. This parameter is not sufficient to select candidates. Indeed, additive, antagonist, or synergistic effects must be considered between pharmacophores included in MTDL. These effects are evaluated in combination assays.

#### 2.5.1. Combination of Two Compounds

The association of drugs can lead to an additive, an antagonist, or a synergistic effect of all drugs involved in vitro or in clinical practice. Additive effects can be produced by equivalent or overlapping actions (retinoic acid and trichostatin A) or independent actions (doxorubicin and trabectedin) [105]. A synergistic effect can be created with several interactions: anti-counteractive actions (cisplatin and topotecan) [106], complementary actions (celecoxib and emodin) [107] or facilitating actions (gentamycin and vancomycin) [108]. Eventually, some drug–drug interactions may modify the efficacy of a mono target by interacting with human pharmacodynamics with additive or antagonistic combinations [22].

To date, no combinations of drugs have been evaluated in clinical trials. This is why we have only considered in vitro assays. All results are presented in Table 7A–C, depending on the methodologies used.

A total of 44 combinations of enterovirus inhibitors were identified. They comprise two classes of inhibitors: direct-acting agents and compounds targeting human-coded protein promoting enterovirus replication.

Two capsid binders (pleconaril and compound **1**) were evaluated in a combination study [109]. Both have different targets in the capsid, and the authors described the synergistic effect of their combination.

Pleconaril also had a strong synergistic effect in combination with other compounds such as rupintrivir, a 3C protein inhibitor, and vemurafenib, an antiproliferative drug [110]. Combination of pleconaril with the 2C protein inhibitor, guanidine HCl, led to a synergistic effect, while pleconaril and oxoglaucine, an autophagy stimulator had an additive effect when combined. At last, the combination of pleconaril and MDL-860, a PI4KIII β inhibitor, had a synergistic effect against CVB3 and an additive one against CVB1 [111,112].

MDL-860 had different synergistic effects depending on the targeted type of virus. Indeed, against CVB1, combinations with either guanidine. HCl or oxoglaucine led to an additive effect, while against CVB3, respectively synergistic and strong synergistic effects were observed [111,112].

Arildone, another capsid binder, had strong synergistic combinations with PTU-23, a 37S and 20S (RF) RNA inhibitor, and HBB, an RNA-dependent RNA polymerase (RdRp) inhibitor [111,112]. On the contrary, arildone and enviroxime, a 3A inhibitor, led to an antagonistic effect when combined.

Disoxaril is also a capsid binder evaluated in several combination assays [111,112]. Combined with PTU-23 or HBB, it led to a strong synergistic effect too, while the combination of disoxaril and enviroxime possessed a synergistic effect.

This last compound, enviroxime, had a strong synergistic effect when combined with PTU-23, HBB, and S-7, which prevents viral uncoating [111,112,113]. Likewise, combinations with HBB and either PTU-23 or S-7 had a strong synergistic effect [111,112].

Furthermore, 3C protease inhibitors have also been evaluated in combination assays.

Rupintrivir, like pleconaril, possessed a strong synergistic effect combined with vemurafenib [110]. Likewise, rupintrivir and interferon-α seemed to lead to a strong synergistic effect [114]. A combination of rupintrivir and cycloheximide, a 60 S subunit of eukaryotic ribosome inhibitor, led to a synergistic effect [110]. However, vemurafenib and cycloheximide were too cytotoxic to be considered a human application. Other compounds, like remdesivir (RdRp inhibitor), dalbavancin (cell well synthesis and anchoring mechanism inhibitor), or anisomycin (a ribosome protein inhibitor), had a lower synergistic effect combined with rupintrivir [110]. When emetine, an NF-κB inhibitor, and rupintrivir were combined, we observed an additive effect [110]. At last, combinations with digoxin (parasympathic system stimulator), homoharrigtonine (anticancer drug), halofuginone (anticancer drug), obatoclax (Bcl-2 inhibitor), or gemcitabine (nucleoside analog) led to antagonistic effect [110].

V-7404, another 3C protease inhibitor, similarly had a strong synergistic effect with capsid binders, pocapavir and vapendavir [115].

However, when we combined rupintrivir and suramin, a capsid inhibitor that binds with the vertex of the fivefold axis, only an additive effect was observed [116]. The combination of capsid binder targeting the hydrophobic pocket and 3C protease inhibitor led to a strong synergistic effect, regardless of the compounds used.

Some combination assays were performed including other compounds against enteroviruses.

Suramin has been evaluated in combination assays with other compounds [116]. Combined with favipiravir, an RdRp inhibitor, a strong synergistic effect was observed, while a combination with itraconazole, a 14α-demethylase inhibitor, led to an antagonistic effect. Both favipiravir and itraconazole, had a strong synergistic effect when they were combined with rupintrivir, but they had antagonistic effects between themselves [116]. Itraconazole was also combined with GW5074, a c-RAF inhibitor, and an antagonistic effect was observed [116].

BPROZ-194, a PI4KIII β inhibitor, also had a synergistic effect with vapendavir [117].

The early stage of enterovirus replication inhibitor YZ-LY-0 was combined with NITD008, an RdRp inhibitor, and GPP3, a capsid binder [118]. Their combinations respectively led to antagonistic and synergistic effects. The antagonistic effect appeared to be inversely proportional to the concentration of YZ-LY-0, while the synergistic effect did not appear to vary greatly with this concentration. Both NITD008 and GPP3 had a strong synergistic effect when they were combined with NK-1.8k [119].

Eventually, likewise, the synergistic effect of the gemcitabine and ribavirin combination seemed to be favored when the gemcitabine‘s concentration increased [120].

**Table 7:** (**A**) Synergistic effect of several inhibitors, measured by MacSynergy II synergistic effect [µM^2^%] *. (**B**) Synergistic effect of several inhibitors, measured by SynergyFinder ZIP score *. (**C**) Synergistic effect of gemcitabine with several concentration of ribavirin, measured by CompuSyn combination index.

(A)																
Synergistic Effect [µM^2^;%] ^‡^		Virus	Pocapavir (V-073)	Vapendavir (BTA798)	Pleconaril	Disoxaril	Arildone	Enviroxime	HBB	MDL-860	NITD008	GPP3	Itraconazole	Favipiravir	Suramin	Ref.
**AG-7404/** **V-7404**		PV Sabin 1	580	463	/	/	/	/	/	/	/	/	/	/	/	[115]
		PV Sabin 2	459	245	/	/	/	/	/	/	/	/	/	/	/	
		PV Sabin 3	288	579	/	/	/	/	/	/	/	/	/	/	/	
**MDL-860**		CVB1 Connecticut	/	/	22.5	/	/	/	/	/	/	/	/	/	/	[111,112]
		CVB3 Woodruff	/	/	61.1	/	/	/	/	/	/	/	/	/	/	
**Guanidine.HCl**		CVB1 Connecticut	/	/	58.4	/	/	/	/	47.5	/	/	/	/	/	
		CVB3 Nancy	/	/	/	/	/	/	/	1.1	/	/	/	/	/	
		CVB3 Woodruff	/	/	56.2	/	/	/	/	83.9	/	/	/	/	/	
**Oxoglaucine**		CVB1 Connecticut	/	/	27.0	/	/	/	/	7.8	/	/	/	/	/	
		CVB3 Nancy	/	/	/	/	/	/	/	220.6	/	/	/	/	/	
		CVB3 Woodruff	/	/	18.6	/	/	/	/	151.0	/	/	/	/	/	
**Enviroxime**		CVB1 Connecticut	/	/	/	82	−39 ^a^	/	253	/	/	/	/	/	/	
**PTU-23**		CVB1 Connecticut	/	/	/	314	519	373	275	/	/	/	/	/	/	
**HBB**		CVB1 Connecticut	/	/	/	689	852	253	/	/	/	/	/	/	/	
**S-7**		CVB1 Connecticut	/	/	/	/	/	399	368	/	/	/	/	/	/	
**Compound 1**		CVB4 Edwards	/	/	147	/	/	/	/	/	/	/	/	/	/	[109]
**YZ-LY-0**	0.125 µM	EV-A71 SK-EV006	/	/	/	/	/	/	/	/	−400–−300	0–100	/	/	/	[118]
	1 µM		/	/	/	/	/	/	/	/	−100–0	0–100	/	/	/	
**Rupintrivir**		EV-A71 FY573	/	/	/	/	/	/	/	/	/	/	450.64	438.07	4.96	[116]
**Favipiravir**		EV-A71 FY573	/	/	/	/	/	/	/	/	/	/	−88.11	/	337.59	
**Suramin**		EV-A71 FY573	/	/	/	/	/	/	/	/	/	/	−246.23	337.59	/	
**GW5074**		EV-A71 FY573	/	/	/	/	/	/	/	/	/	/	−167.68	/	/	
**(B)**																
**ZIP Synergy Score [−30/30] ^†^**			**Virus**			**Pleconaril**			**Rupintrivir**					**Ref.**		
**Rupintrivir**			E1 Farouk (A549 cells)			18.0			/					[110]		
			E1 Farouk (RPE cells)			18.9			/							
**Vemurafenib**			E1 Farouk (A549 cells)			13.6			15.6/16.00							
			E1 Farouk (RPE cells)			20.2			17.2							
**Pleconaril**			E1 Farouk (A549 cells)			18.0/18.3			18.0/18.3							
			E1 Farouk (RPE cells)			18.9			18.9							
**Cycloheximide**			E1 Farouk (A549 cells)			/			10.7							
**Remdesivir**			E1 Farouk (A549 cells)			/			8.23							
**Dalbavancin**			E1 Farouk (A549 cells)			/			8							
**Anisomycin**			E1 Farouk (A549 cells)			/			6.39							
**Emetine**			E1 Farouk (A549 cells)			/			4.1							
**Digoxin**			E1 Farouk (A549 cells)			/			−5.6							
**Homoharringtonine**			E1 Farouk (A549 cells)			/			−5.7							
**Halofuginone**			E1 Farouk (A549 cells)			/			−7.5							
**Obatoclax**			E1 Farouk (A549 cells)			/			−7.8							
**Gemcitabine**			E1 Farouk (A549 cells)			/			−8.7							
**(C)**																
**Concentration of Ribavirin (µM)**						**25**		**50**			**100**	**200**	**400**	**Ref.**		
**Combination Index with 0.4 µM of gemcitabine °** **(CVB3 on Vero cells)**						0.28		0.23			0.15	0.14	0.14	[120]		

(A) * Compounds evaluated in clinical assays have their rows and columns shaded. ^‡^ Synergistic effect (SE) is calculated using the MacSynergy II software. It measures strong synergistic effect (if SE > 100 µM^2^%), synergistic effect (if 50 µM^2^% < SE < 100 µM^2^%), additive effect (if 0 < SE < 50 µM^2^%) and antagonistic effect (if SE < 0 µM^2^%). ^a^ Combination of enviroxime and arildone led to an antagonism effect measured at −39 µM^2^%. (B) * Compounds evaluated in clinical assays have their rows and columns shaded. ^†^ ZIP Score is calculated using SynergyFinder version 3 software. It measures strong synergistic effect (if ZIP Score > 15), synergistic effect (if 5 < ZIP Score < 15), additive effect (if −5 < ZIP Score < 5) and antagonistic effect (if ZIP Score < −5). (C) ° Combination Index (CI) is calculated using CompuSyn software (https://compusyn.software.informer.com/, accessed on 30 June 2024). It measures synergistic effect if it’s inferior to 1 (CI < I), additive effect if equal to 1 (CI = 1), antagonist effect if superior to 1 (CI > 1).

#### 2.5.2. Combination of Three Compounds

Some assays were performed by combining three compounds in vitro to improve the efficacy of compound combinations and avoid viral resistance.

Some triple consecutive alternating administration (CAA) combination assays were performed by the Galabov Bulgarian team, namely one drug per day in 3-day cycles. They combined disoxaril, guanidine hydrochloride, and oxoglaucine (DGO) against CVB1 and CVB3 [121,122,123,124]. Other CAA combination studies were led by the same team substituting disoxaril with pleconaril (PGO combination) or guanidine-hydrochloride by MDL-860 (PMO combination) against the same viruses [121,122,123,124,125,126,127]. These first assays were performed to decrease the resistance established with a combination of disoxaril and enviroxime [128]. In this type of experiment, order of administration seems essential: first, a capsid binder, then a 2C protease inhibitor, and finally, an active compound in the early stage of viral replication. Other orders of administration showed less or no inhibition of viral proliferation. Likewise, adding PTU-23 to make a 4-day cycle suppresses the antiviral potency. DGO combinations are more effective than PGO combinations. Substituting guanidine-hydrochloride (45 mg/kg) by MDL-860 (75 mg/kg) increases the anti-enteroviral effect. The concentration of MDL-860 should be compulsory to maintain the potency without developing toxicity due to higher doses. CAA combinations keep the potency of the most effective drugs among the three inhibitors. Moreover, combination with CAA should reduce the risk of resistance apparition but also increase drug sensitivity in comparison with every drug treatment used alone or administrated simultaneously. This is an exciting approach, but it remains to be seen whether it is effective and can be implemented in humans, where the time of diagnosis does not necessarily coincide with the onset of enteroviral infection.

Recently, a new study combined remdesivir (RNA-dependent-RNA-polymerase inhibitor), rupintrivir (3C protease), and pleconaril (capsid binder) with a higher in vitro efficacy than monotherapy or two-drug cocktail [129]. This combination was successfully evaluated on a broad spectrum of enterovirus, including five coxsackieviruses, three echoviruses, encephalomyocarditis virus, EV-A71, EV-D68, foot-and-mouth viruses, three polioviruses, and nine rhinoviruses. Moreover, this three-drug cocktail also delayed the emergence of antiviral drug resistance for any compound in the combination.

Even if clinical trials of capsid binders were stopped, the hydrophobic pocket inside the VP1 capsid canyon remains the easiest target with conserved domains between species (except for RV-C) [112,113,114]. However, non-structural proteins are also prime targets for the weak fluctuation of their constitution between species, even if double-membrane vesicles protect them.

An MTDL strategy is based on combination therapies, which result in a synergistic effect with only one compound, not a cocktail of drugs. According to the most recent combination therapies tested, capsid binders have a strong synergistic effect with 3C protease inhibitors and a moderate or weak effect with 3A protease inhibitors. PI4KIIIβ inhibitors also have an additive synergy with capsid binders.

#### 2.5.3. Other Criteria

The degree of resistance can determine for the selection of candidates for broad-spectrum enterovirus inhibitors. For instance, pleconaril treatment confronts some drug resistance in some clinical trials, with a part of natural resistance [54,130]. Eventually, the selectivity of viral targets by inhibitors is also important to reduce drug-emergent adverse effects due to binding with unexpected human proteins.

### 2.6. Biological Parameters for MTDL Strategy

#### 2.6.1. Enteroviruses of Interest

Determining which EVs should be included in the MTDL concept strategy requires analysis of pathogenesis, epidemiology and evolution, surveillance program, and already-market therapeutic options [131]. Considering all these points, we have selected non-polio enteroviruses (NPEVs), including important human pathogens, such as coxsackieviruses, echoviruses, numbered enteroviruses, and rhinoviruses [22].

Infections related to these viruses have been described frequently over the last decade. We first noted clusters of infection, followed by a global emergence for EV species, specifically, EV-A-71 in the Asia-Pacific region and EV-D-68 in North America and Europe. Rhinoviruses (RV) epidemiologic studies classified these viruses as the most frequent infectious agents in humans worldwide [132,133,134].

Finally, considering more than one virus species in a large-scale program of medicinal chemistry is pertinent if these pathogens are detected in the same type of disease or isolated from the same clinical samples. Moreover, the drug candidate must exert a broad-spectrum activity, which, to our knowledge, is the case for several viruses of this genus.

#### 2.6.2. Biological Access to the Target

Enteroviruses are obligatory intracellular parasites that need host cells to replicate. Externally accessible targets are mainly situated on the viral capsid. Likewise, capsid binders targeting hydrophobic pockets can interact with their target in the extracellular viral cycle sequence. Sometimes, the pocket is empty, like in HVR-B14, but mostly, is filled with a fatty acid or a fatty acid derivative (lipped fatty acid or sphingosine), and called a pocket factor [135].

For inhibitors targeting viral proteins or proteases, the first main barrier remains the cellular membrane after entering the cell before inhibiting viral replication. Moreover, it is known that ssRNA (+RNA) viruses remodel intracellular membranes to support viral replication. Enteroviruses make closed single-membrane tubules and then double-membrane vesicles to escape the surveillance of the cell’s immune system [136,137,138,139].

Consequently, inhibitors targeting viral non-structural proteins may have more difficulty accessing their targets than inhibitors of viral structural proteins. However, any target will be privileged to meet our objective of proposing an MTDL candidate. Thus, the pharmacokinetic parameters of MTDL candidates are critical and may need refinement in the function of the target.

These limitations can be applied to one drug, drug cocktails, or MTDL treatments.

#### 2.6.3. Conserved Residues of Targets—Antiviral Resistance

Some features within the capsid are highly conserved for most RV species. For example, the hydrophobic pocket sequence has a high degree of amino acid identity for both RV-A and RV-B species [140]. Most recently, this remaining identity has also been found in EV-A71 and all other types of the enterovirus genus [141]. These results are due to the proximity between the hydrophobic pocket and the VP1-VP3 interface. Amino acids near the interface between viral proteins are the most conserved from one serotype to another.

Differences between protein or protease coding sequences are rare for the rest of the viral genome, like in species EV-A [142]. Indeed, mutations of amino acids in these scaffolds should lead to irreversible changes in the recognition of viral substrates, even if some were detected after in vitro mutation assays [59,143,144,145,146,147,148,149,150].

One of the risks of using antivirals is the emergence of resistance, which results in reduced or loss of activity. This phenomenon has been observed for capsid-binding inhibitors after numerous in vitro expositions [5,19,143].

## 3. Discussion

As any anti-enteroviral was refused to be commercialized due to several clinical trials failing of evaluated compounds, can their lack of efficacy and adverse effects be addressed with multi-target directed ligands (MTDLs)?

We described 51 clinical trials included in the analysis according to our inclusion criteria.

The design of the studies is based either on a human subject virus challenge using ethically approved infection pathways, with a known treatment initiation time and virus type or on patients included and randomized after the description of clinical signs of infection (the type is inherent to each trial). The treatment was usually administered the day or a few hours before the virus challenge; more rarely after inoculation. In the case of naturally-occurring infections, several cases were noted: either the treatment time is as short as possible (24 or 48 h after the first symptoms), or it is a last-line treatment with no delay given the context of care (compassionate use). The time for initiation of treatment is sometimes absent. The fact of not having details on the time between the appearance of the first symptoms and the initiation of treatment is problematic. In fact, in acute infections, the effects of antiviral therapies strongly depend on the early administration of the antiviral treatment. In the case of enteroviruses that have a fast replication time, a short time for the initiation is decisive. Hence, the results of trials need to be evaluated based on the time of therapy from the earliest symptoms/signs of infection. We must take this information into account when prioritizing compounds to include in an MTDL approach.

Moreover, in the case of naturally occurring infections, the effectiveness of the treatment is evaluated on the circulating virus. This element can give more weight to the results of these clinical trials considering the difference that can be observed between the assessments in the experimental conditions and the studies in real-life conditions.

With the aim of discussing the MTDL concept, we presented the efficacy data of in vitro multitherapy. Here, a few class associations have been described as synergistic. We noted that not all compounds in the same class achieved the same result in association with another compound.

### 3.1. Multi-Target Directed Ligand (MTDL) Definition

**Figure 7:**
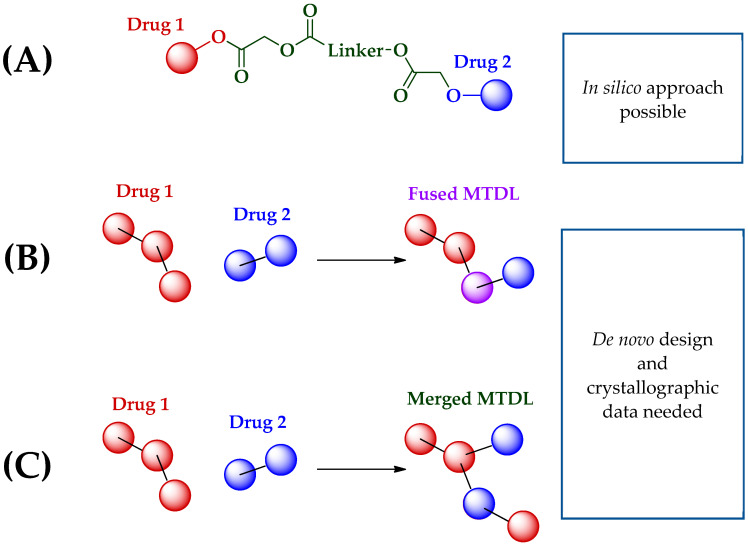
Illustration of concept or MTDL. Multitarget-directed ligands (MTDLs) can be classified as “linked” (**A**) or “fused” (**B**) or “merged” (**C**) based on the degree of overlap of the pharmacophores.

MTDLs combine pharmacophores from different inhibitors to treat multifactorial diseases, here, targeting several enterovirus (EV) proteins with one compound. They are drugs containing two or more pharmacophores structurally overlapping or separated by a spacer in a single molecule. The linked molecules comprised an enzyme-cleavable linker binding the active inhibitors, with minimal alteration to their initial structure. Their design can include the fusion of compounds that happens when one chemical group is identified in two drugs, which are fused by this group to obtain one only compound, while merging has the same concept, not focusing on chemical structure but pharmacophores’ interactions. These concepts are illustrated in Figure 7.

To ensure that affinity with the various targets is preserved, in silico evaluation is required. Fused or merged MTDL must be supported with de novo and crystallographic data while compounds bound by a labile linker can be designed with an experimental approach only as they are released with their initial form. In the design-in approach, the fusion or merge must preserve interactions of inhibitors with each target. It enables us to determine whether scaffold hopping is workable and whether substituents are feasible. Design-out begins with a compound acting simultaneously on all targets, which is not our case here.

**Figure 8:**
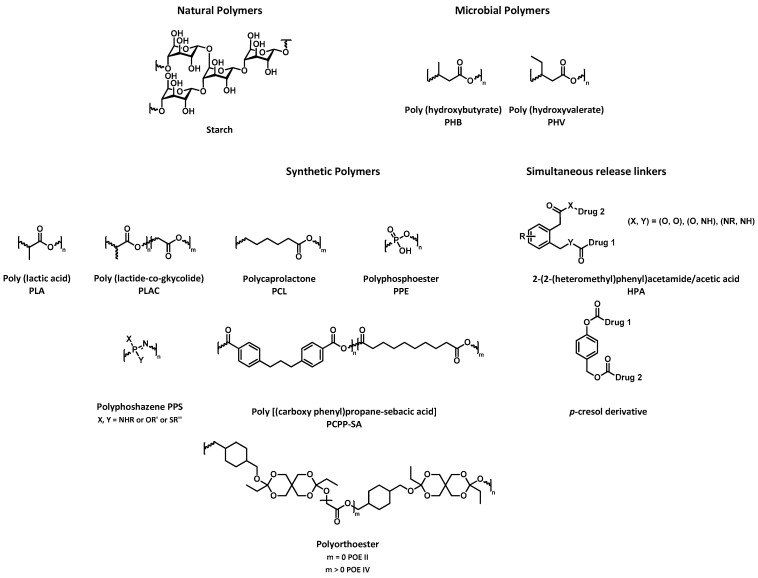
Enzyme degradable linkers.

Fusion of several chemical functions or merging of numerous pharmacophores requires adapting synthesis pathways for inhibitors. In this case, the maintenance of the affinity of the new compound for each considered target is challenging. The use of a labile linker allows compounds to be released in vivo and could limit the loss of affinity for the respective targets. Here, the key parameter is the release of each compound. Enzymatic cleavable binding agents are well known, including their structure [Figure 8] and degradation rate [151]. New linkers have recently been developed to perform simultaneous versions, while traditional ones run them sequentially.

### 3.2. MTLD Challenges and Alternatives

The major challenge of MTDLs is to overcome the lack of efficacy of compounds, evaluated in clinical trials. They must also maintain the pharmacokinetic parameters of initial inhibitors and enhance their selectivity. Moreover, long treatment with MTDL doesn’t have to lead to drug-induced resistance.

In addition, there are other alternatives to small organic molecules. Indeed, some biological entry inhibitors have had their efficacy assessed in vitro and in vivo assays. For instance, therapeutic antibodies have been developed and validated in preclinical models [152,153]. However, they may be too specific for a reference virus type and ineffective for other viral strains from the same type [154]. Peptides and membrane receptors were also used against enteroviruses [155,156]. However, their development dates to the 2000s, with no further progress thereafter.

### 3.3. Benefit–Risk Balance of MTDL

Thus, the efficacy of the drug can be multiplied as an MTDL rather than a single molecule. Indeed, MTDL mimics drug combinations, including additive or synergistic effects. With well-associated combinations, synergy can increase benefit–risk balance and favor the success of clinical trial studies. Research on this topic spans various disease applications, including Alzheimer’s [157,158], cancer, and infectious diseases. Understanding drug resistance in cancer supports the development of MTDLs, coupled with clinical experience, which has revealed a multi-target strategy to enhance anticancer activity [159]. This approach holds significant potential in fighting infectious diseases [160,161,162,163,164,165,166]. In virology, multi-target compounds have been developed for pulmonary diseases targeting F508del-CFTR and PI4KIIIβ [167]. This approach is fascinating as it simultaneously inhibits viral replication and reduces the intensity of symptoms [168]. Virtual screening was recently performed in SARS-CoV-2, CVB1, CVB3, and E1 treatment research with the goal of designing multi-target compounds [102,118,169]. Many projects described above were designated for in silico evaluation of the interest of linked, merged, or fused compounds to produce an effective compound library [170,171].

Several combinations can increase the potency of enterovirus inhibitors to achieve a broad-spectrum effect against non-polio enteroviruses (NPEVs) or enhance antiviral potency. A broad-spectrum approach is interesting because most NPEVs have similar mild clinical manifestations with different complications. Having a single compound for every NPEV eliminates the need for virus identification, leading to more effective patient management. This advantage is crucial for the patient, as therapy initiation can be prompt without the time-consuming process of viral typing, which requires specialized equipment. Fusing, merging, or linking several inhibitors in one MTDL leads to a need for a balance between the affinity of the MTDL with its different targets and its pharmacokinetic parameters.

The single-chemical approach utilized by MTDLs can reduce adverse effects and enhance pharmacokinetic/pharmacodynamics parameters compared to combinations of individual single-target drugs [157,158]. However, MTDLs based on a linker to bind selected candidates can have a high molecular weight. Formulation or transport studies must be performed to evaluate the bioavailability of synthesized new MTDLs. Macromolecules, polymers, or pharmacokinetic enhancers can be added to improve the passage through the intestine barrier.

This approach may also lead to a more effective treatment, having additive or synergistic effects by targeting several viral proteins. Compounds, before linking, fusion, or merging must have synergistic effects between them. Some bindings can be modified for fused or merged MTDLs. Indeed, including partial structure or pharmacophore of initial inhibitor in one compound could lead to adverse interactions or steric hindrance with the targeted viral proteins. These effects could decrease the absolute efficacy of MTDL without considering synergistic effects. Thus, MTDL should modify only the structure without affecting the binding mode of the initial inhibitor. MTDLs, gathering several initial inhibitors, generally have a higher molecule weight that implies a better specificity to target considered viral proteins. MTDLs try to maintain the same binding mode with the target but do not have the same interactions with unintentional cell metabolism steps. Consequently, it automatically decreases the adverse effects linked with these steps [157,158].

Moreover, the MTDL strategy delayed the emergence of drug-related resistance. Viruses can develop lower sensitivity due to multiple contacts with the drug in monotherapy or lower doses of inhibitors in combinations [146]. Having one merged or fused compound targeting several parts of the virus, the plurality of targets, enables delaying the onset of resistance due to acquired mutations, as well as maintaining higher concentrations deleting drug–drug interactions present in classic combinations. Even with enzyme-cleavable linked drugs, MTDL release can be controlled to avoid these issues.

MTDL design is also easier to conduct than combinations due to fewer parameters to evaluate, even in comparison with single-target drug development [157,158]. Thus, MTDL design should have reduced cost development with superior antiviral activity.

Eventually, MTDL pharmacokinetic parameters are easier to manage than a combination strategy [157,158]. Different initial drugs gathered in MTDL do not need the same bioavailability, pharmacokinetics, or metabolism to form an MTDL. Contrary to MTDL, combination therapy must apply this criterion to be effective, with the same pharmaceutical form and pharmacodynamics, and avoid drug–drug interactions.

### 3.4. MTDL Proposal

**Figure 9:**
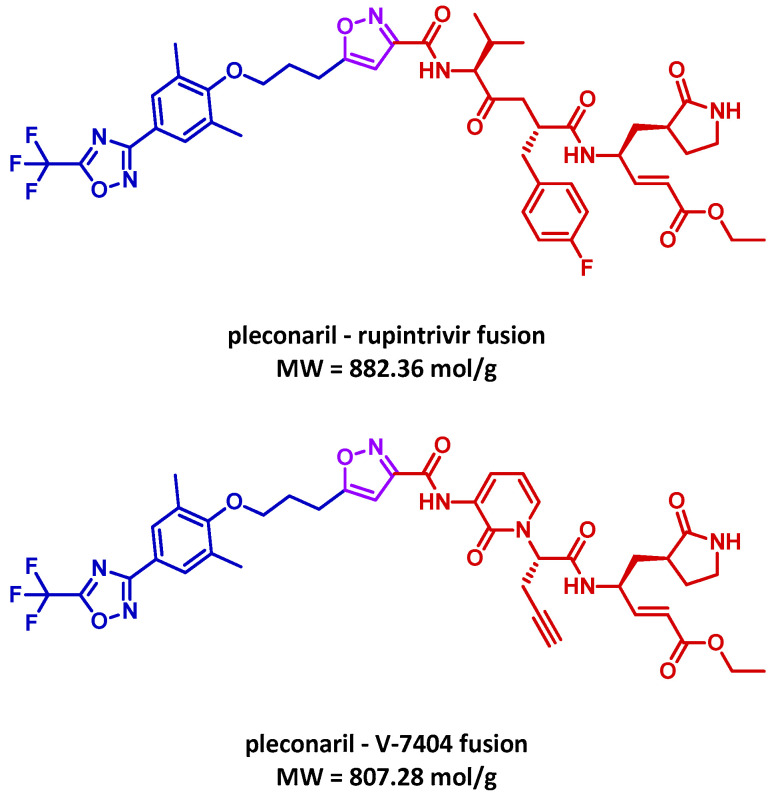
Proposals of MTDL combining pleconaril (in blue) and rupintrivir or V-7404 (in red). Common isoxazole in presented in purple.

In our presentation of inhibitor structures, we identified chemical groups that can be used to fuse or link several compounds to form MTDLs. This recognition of certain chemical functions remains partial and only claims to encompass some of the possibilities offered by these molecules. It is necessary to have carried out in silico studies and design de novo to avoid speculative combinations and “no druggable” compounds. For example, according to our results, a combination of capsid binder and 3C protease inhibitors seems to be strongly promising. Pleconaril and rupintrivir or V-7404 have broad-spectrum activity. All these inhibitors present an isoxazole. However, fusion by this chemical group leads to heavy compounds (molecular weight > 800 Da) [Figure 9].

## 4. Materials and Methods

We chose to limit this study to NPEV, excluding poliovirus inhibitors. The commercially available poliomyelitis virus vaccine is effective in preventing PV infection whereas there is no vaccine for all types of NPEV (except for EV-A71 in China). In this review, we summarize recent insights from NPEV research considering in vivo efficacy data described in clinical data.

### 4.1. Literature Survey

The literature survey was conducted using the website Clinicaltrials.gov (CT), the European Union Drug Regulating Authorities Clinical Trials Database (EudraCT), the International Clinical Trials Registry Platform (ICTRP), and PubMed. The data collection period occurred in June 2024. The search parameters included every clinical trial written in English. The keywords used for study inclusion were enterovirus (namely, “Coxsackievirus”, “Echovirus”, “Enterovirus”, “Enterovirus Infections”, or “Rhinovirus). The exclusion criteria were clinical trials on vaccines, devices, or biological drugs. For the combination part, the literature survey was conducted on PubMed only, using keywords “combination” and “Enterovirus”.

The type and name of the inhibitor, its chemical structure, studied type of EV infection or disease, concentration, doses, and pharmaceutical form for drug delivery, parameters of cohorts (age, gender), and conclusions of studies were used for reporting.

The Prisma flowchart has been adapted to classify the data and identify studies for our analysis. The details of the items are presented with the main results of our research in the results section, Scheme 1 [21].

### 4.2. MTDL Candidate Proposal

The two data types used to discuss an MTDL strategy were compound parameters (broad-spectrum activity, target selectivity, effect during in vitro combination, identified mechanism of action) and virus characteristics (interest in virus, viral resistance, target’s interest, and knowledge of the target).

We discuss the interest of each association and analyze molecule properties to propose an optimized MTDL with higher potency and the most favorable PK/PD parameters.

MTDLs are commonly designed according to three categories. The least invasive method is to link several inhibitors, which have been studied in combination therapy. Another pathway consists of fusing similar structures in two compounds to synthesize only one compound. Finally, some inhibitors with the same pharmacophoric elements can be merged into one inhibitor. To optimize MTDL, initial compounds must have similar activity and PK/PD or physicochemical parameters to keep the same Absorption, Distribution, Metabolism, Excretion (ADME) profile and have similar levels of doses to be administered.

## 5. Conclusions

In this study, we reviewed clinical trials that evaluated inhibitors of enterovirus and classified them according to their target. Viral structural protein inhibitors only target VP1 capsid protein. 3A/3AB and 3C proteases are viral non-structural proteins. Several human proteins (cholinergic receptors, phosphatidylinositol 4-kinase β PI4KB or α-adrenoreceptors) were studied as potential anti-enteroviral targets as well as some broad-spectrum antiviral, anti-rhinovirus inhibitors without defined mechanism of actions, and other class of drugs (anti-asthmatic and antibiotics). Non-structural proteins have a low mutation rate and are conserved between each enterovirus serotype. However, synthesizing double-membrane vesicles, protecting viral genetic material during translation or replication, makes them more challenging to access. On the other hand, capsid seems more straightforward to access. Moreover, the capsid hydrophobic pocket also has a conserved sequence, except for RV-C serotypes. According to combination assays described in the literature, targets to select to have the best efficacy are capsid VP1, 3C protease, and a host target, like PI4KIIIβ. However, even if enterovirus inhibitors were well studied, none were commercialized due to a lack of benefit in the efficacy–risk–cost balance. This perspective proposes MTDL as a solution to overcome these obstacles. They enable enhanced efficacy with additive or synergistic effects. Likewise, it allows the reduction of opportunities for resistance to appear and limits drug-emerged adverse effects. Moreover, with fewer parameters to evaluate, MTDL assays are more accessible to develop rather than cocktail combination clinical trials. However, using linked, fused, or merged MTDL, the pharmacokinetic parameters and binding modifications must be considered to obtain an optimized broad-spectrum enterovirus inhibitor.

## Data Availability

Data sharing is not applicable.
